# Research on Microwave Pyrolysis Recovery and Reuse Performance of Carbon Fiber Composites

**DOI:** 10.3390/polym16101383

**Published:** 2024-05-12

**Authors:** Xuan Li, Lei Xu, Yiyao Ren, Zheng Nan, Shijie Xiao, Zhigang Shen

**Affiliations:** 1 Faculty of Metallurgical and Energy Engineering, Kunming University of Science and Technology, Kunming 650093, China; chn_lx0214@126.com (X.L.); renyy7616@163.com (Y.R.); nanz255@163.com (Z.N.); 2 State Key Laboratory of Complex Nonferrous Metal Resources Clean Utilization, Kunming University of Science and Technology, Kunming 650093, China; 3 Key Laboratory of Unconventional Metallurgy, Ministry of Education, Kunming University of Science and Technology, Kunming 650093, China; 4 SINOPEC Shanghai Research Institute of Petrochemical Technology Co., Ltd., Shanghai 201208, China; xiaosj.sshy@sinopec.com

**Keywords:** recovery, carbon fiber, reinforced composites, mechanical property

## Abstract

Carbon fiber-reinforced resin matrix composites find extensive applications across various industries. However, their widespread use also generates significant waste, leading to resource depletion and environmental concerns. Studying the production of composite materials using recovered carbon fiber is imperative to mitigate the environmental impact associated with waste from carbon fiber-reinforced resin matrix composites and optimize resource utilization. In this study, carbon fiber was reclaimed using the microwave pyrolysis–oxidation process. The reclaimed carbon fiber underwent a cutting process to produce shorter carbon fibers tailored to specific requirements, which were then used to fabricate composite plates reinforced with epoxy resin. The mechanical characteristics of the composite were analyzed, along with SEM, XPS, infrared, Raman, and contact angle analyses conducted on the recovered carbon fiber. The test findings suggested minimal variation in the surface morphology of the recovered carbon fiber materials. Post-recovery, an increase in the quantity of oxygen-containing functional groups was observed on the carbon fiber surface. Additionally, the contact angle between the carbon fiber surface and the epoxy adhesive decreased. The mechanical properties of the composite produced from the recovered carbon fiber decreased, including the impact strength, tensile strength, and bending strength, with the impact strength dropping by 24.14%, tensile strength by 15.94%, and bending strength by 8.24%, while maintaining overall reusability, thus paving the way for the comprehensive utilization of carbon fiber resources.

## 1. Introduction

Carbon fiber-reinforced resin matrix composites [1,2] are extensively utilized across various industries, including aviation, aerospace, and defense, due to their exceptional characteristics such as superior specific strength, specific modulus, lightweight nature, fatigue resistance, and customizable design features. However, the growing utilization of these composite materials contributes to a significant volume of waste generated from cutting and end-of-service components [3]. Since carbon fiber composites are not biodegradable, their disposal poses environmental challenges and leads to substantial waste of carbon fiber resources [4]. Additionally, the production of carbon fiber materials requires substantial energy consumption [5], resulting in high manufacturing expenses. Consequently, the recovery of carbon fiber composites has become a significant focus of study in materials science.

Currently, research on the application of recovered carbon fiber has yielded significant results. A research team at the Tokyo University of Science in Japan has developed an efficient process for preparing recycled carbon fibers, achieving highly pure recovered fibers suitable for applications in the aerospace and automotive industries [6]. The Circular Materials Innovation Centre in the UK is also dedicated to research and development in the field of recovered carbon fiber. Collaborating with industries, they provide solutions and support for recycled carbon fiber using various recovery processes and technologies [7]. Similarly, research institutions such as the University of Pennsylvania [8], Michigan State University [9], and others in the United States have conducted extensive research on the preparation and application of recovered carbon fiber. They have explored diverse regeneration processes and methods to optimize fiber structure, aiming to enhance the performance and sustainability of recovered carbon fiber [10]. In Canada and Australia, research teams are also focusing on recycled carbon fiber, refining the regeneration process, analyzing the performance, and expanding its application fields [11]. The utilization of recovered carbon fiber in composite materials preparation offers a cost advantage. Kravtsova et al. discovered the feasibility of recovering thermoplastic fiber-reinforced composites through thermoforming, ensuring material availability despite a decrease in mechanical properties after multiple recycling processes [12]. Current research actively advocates for the reutilization of recovered carbon fiber, emphasizing its physical and chemical properties, microstructure, and other relevant aspects. Through technical enhancements, process optimization, and other methods, the performance and application potential of recovered carbon fiber and its composite materials can be enhanced, offering a novel path for the sustainable advancement of carbon fiber technology.

## 2. Experimental Section

### 2.1. Materials

T300 carbon fiber from Toray Co., Ltd. (Tokyo, Japan) was selected as the reinforcing material for preparing the short-cut carbon fiber-reinforced composite, while EP621 material from Dongguan Fengjin New Materials Co., Ltd. (Dongguan, China) was selected as the dipping resin for the short-cut carbon fiber. The tensile modulus of T300 cut carbon fiber is 219.6 GPa, the tensile strength is 3230.7 MPa, the elongation at break is 1.8%, the density is 1.8 g/cm^2^, and the fiber diameter is 7 μm. The KBr used had a purity of 99.95% and was sourced from Macklin Chemical Reagents Co., Ltd. (Shanghai, China).

The T300 carbon fiber was cut into short fibers approximately 10 mm in length. These short-cut carbon fibers, along with epoxy resin, were used to prepare the short-cut carbon fiber-reinforced resin matrix composite material, using the hot pressing technique (DCGYJ300, Jiangsu DIPUER Industrial Co., Ltd., Wuxi, China). The maximum nominal pressure of this equipment is 3000 KN, with a pressing area of 750 mm × 750 mm, and a temperature range of 50–250 °C. The preparation process proceeded as follows: Firstly, the mold was coated with a release agent on its inner surface, followed by preheating the mold at 70 °C for 20 min. Then, the short-cut carbon fiber and epoxy resin were mixed into the mold according to a mass ratio of 52:48. Subsequently, the short-cut carbon fiber-reinforced resin matrix composite plates were prepared at a curing temperature of 120 °C with a pressing stress of 8 MPa for 40 min.

### 2.2. Experimental Procedure

**Figure 1:**
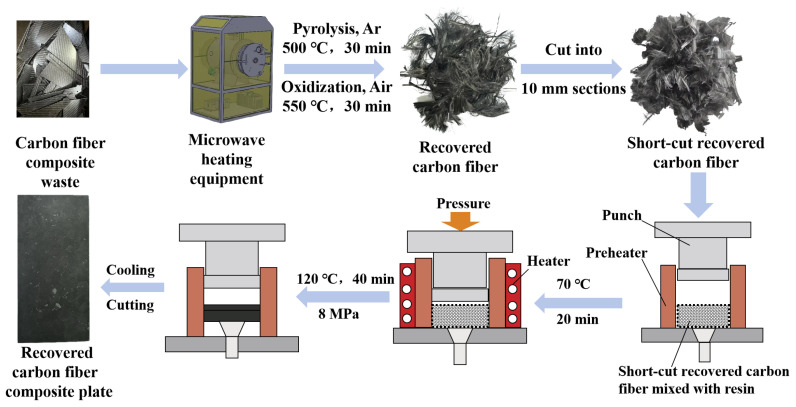
Carbon fiber plate preparation process flow chart.

The recovery of the carbon fiber composite was achieved through the microwave pyrolyzation–oxidation method. The concrete steps were as follows: Firstly, the carbon fiber composite plate, with dimensions of 200 mm × 100 mm × 1 mm, was placed into a boat-shaped mullite crucible, which was then positioned in a microwave tube furnace. Prior to the reaction, argon gas was introduced into the furnace as a safeguarding measure, followed by a brief purging of air. This sequence was repeated three times to ensure the complete elimination of air. Subsequently, argon was introduced into the microwave tube furnace at a flow rate of 50 mL/min, and microwave radiation was applied. The carbon fiber composite was directly heated to 500 °C under microwave radiation at a frequency of 2450 MHz and maintained at this temperature for 30 min. The carbon fiber composite was then heated to 550 °C by air and oxidized for an additional 30 min [13]. After cooling the pyrolysis products to room temperature, they were washed with water and dried at 60 °C for 20 h to obtain recovered carbon fibers. These recovered carbon fibers were then cut into short fibers approximately 10 mm in length. The short-cut recovered carbon fibers were combined with epoxy resin to create a composite material with a resin matrix, using the hot pressing technique (DCGYJ300, Jiangsu DIPUER Industrial Co., Ltd., Wuxi, China). The preparation procedure involved applying a release agent to the inner surface of the mold, followed by preheating the mold at 70 °C for 20 min. The carbon fibers, cut to a length of approximately 10 mm, were then combined with epoxy resin in a mold in a mass ratio of 52:48. The fiber volume content was about 42%. The mixture underwent controlled heating at 120 ℃ for a holding time of 40 min under a pressure of 8 MPa to ensure thorough impregnation of the short-cut carbon fibers. Upon completion of the insulation period, the mold was rapidly cooled to room temperature, followed by demolding and excess material removal through cutting. This process resulted in the production of a board made of recovered carbon fiber-reinforced resin matrix composite material. Subsequently, the tensile, bending, and pendulum impact strength of the composite plates were evaluated, and the fracture surface morphology of the short-cut carbon fiber-reinforced composite was examined. The procedure for preparing the short-cut carbon fiber composite sheet is illustrated in Figure 1.

### 2.3. Characterization Methods

The surface morphology of carbon fiber and the tensile section of the short-cut carbon fiber-reinforced composite before and after recovery were observed using scanning electron microscopy (SEM, TESCAN MIRA LM, Brno, Czech).

Fourier infrared spectroscopy (FT-IR, Nicolet iS50, Thermo-Nicolet, Waltham, MA, USA) was utilized to analyze the functional group changes of carbon fiber before and after recovery. Carbon fiber samples before and after recovery were mixed with KBr and pressed to prepare FT-IR samples. The changes in the infrared spectrum of carbon fiber before and after recovery were observed, and the alterations in the chemical bonds of carbon fiber were analyzed.

X-ray photoelectron spectroscopy (XPS, Thermo Scientific K-Alpha, Thermo Fisher Scientific, Waltham, MA, USA) was employed to examine the elemental composition of carbon fiber both before and after the recovery process, as well as to determine their respective concentrations. The X-ray photoelectron spectrometer adopted the constant analyzer energy mode for XPS testing. The energy of the spectrometer was set to 1591.8 eV, with a working voltage of 20 kV, power of 160 W, and a beam spot size of 500 μm. A Raman spectrum analyzer (alpha300R, WITec, Ulm, Germany) was utilized to analyze the carbon structure in carbon fiber before and after recovery.

Raman spectrum analysis was performed in the constant analyzer energy mode, with a spectrum scanning range of 186~5000 cm^−1^ and an output power of 50 mW.

A contact angle measuring instrument (DSA25S, KRUSS, Hamburg, Germany) was employed to assess the wettability of epoxy resin on carbon fiber surfaces before and after recovery.

According to the ASTM D-3822 standard [14], the mechanical properties of carbon fibers before and after recovery were measured using a universal automatic single fiber physical property analyzer (Textechno FAVIMAT+ROBOT2, Textechno H. Stein, Mönchengladbach, Germany). Initially, the diameter of the carbon fiber was measured using an optical microscope, and from this, the cross-sectional area and density were calculated. Subsequently, a 10 cm length of carbon fiber was secured onto the mechanical frame, and a tensile test was carried out using the testing machine. The moving speed was set to 1mm/min, and both load and elongation data were recorded during the test. From these data, the tensile modulus and tensile strength were calculated. These tests were repeated, with at least 30 tests performed for each sample to obtain the average data.

The pendulum impact strength of composite materials before and after recovery was conducted in accordance with the ASTM D-256 standard [15]. The tests were performed using a pendulum impact testing machine (HIT25P, Zwick Roell, Ulm, Germany), with the pendulum energy set at 5 J. The sample size of the composite impact test before recovery was 60.0 mm × 5.0 mm × 2.8 mm, while, after recovery, it was 60.0 mm × 5.0 mm × 2.4 mm.

The tensile modulus, tensile strength, and elongation at the break of carbon fiber-reinforced composites before and after recovery were conducted in accordance with ASTM D-3039 [16]. These tests were performed using a universal testing machine (CMT6103, Meters Industrial Systems, Eden Prairie, MN, USA). The specifications of carbon fiber-reinforced composite materials before and after recovery were 100.0 mm × 10.0 mm × 2.7 mm and 100.0 mm × 10.0 mm × 2.4 mm, respectively. The original standard distance was 80 mm. The tensile tests were conducted at a drawing speed of 2 mm/min at room temperature. At least 5 specimens for each type were tested in this study.

The bending strength and bending modulus of carbon fiber-reinforced composites before and after recovery were conducted in accordance with ASTM D-790 [17]. These tests were performed using a universal testing machine (CMT6103, Meters Industrial Systems, USA). The specifications of carbon fiber-reinforced composites before and after recovery were 80.0 mm × 10.0 mm × 2.7 mm and 80.0 mm × 10.0 mm × 2.4 mm, respectively. The sample span was set to 64 mm. The bending tests were conducted at a constant speed of 0.2 mm/min at room temperature. At least 5 specimens for each type were tested in this study.

## 3. Results and Discussion

### 3.1. Properties of Recovered Carbon Fiber

**Figure 2:**
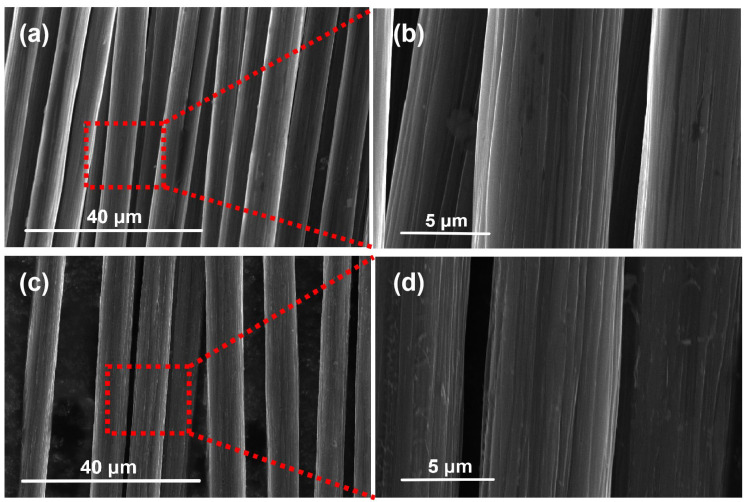
(**a**,**b**) SEM results of carbon fiber before recovery, (**c**,**d**) SEM results of recovered carbon fiber.

Scanning electron microscopy (SEM) was conducted on the carbon fiber before and after recovery to observe the surface morphology changes in the recovered carbon fiber. The results are depicted in Figure 2. Figure 2a,b illustrate the micromorphologies of virgin carbon fiber, while Figure 2c,d represent the micromorphologies of the recovered carbon fiber [18]. The microstructure difference between the carbon filaments before and after recovery is minimal. A small amount of carbon and resin remains on the surface, and no obvious defects are found. The grooving structure is retained, indicating that the carbon fiber is not significantly damaged during the recovery process and exhibits good recovery performance [19].

**Figure 3:**
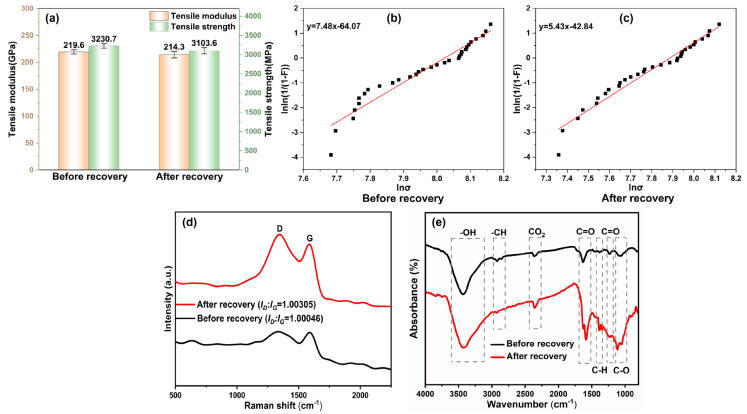
(**a**) Tensile test results of carbon fiber monofilament before and after recovery, (**b**) Weibull distribution of before recovery fiber tensile strengths, (**c**) Weibull distribution of after recovery fiber tensile strengths, (**d**) Raman test results of carbon fiber before and after recovery, and (**e**) infrared test results of carbon fiber before and after recovery.

The purpose of the test was to evaluate the tensile strength and tensile modulus of a carbon fiber monofilament both before and after the recovery process. The findings, illustrated in Figure 3a, demonstrate that the tensile strength and modulus of the carbon fiber monofilament were 3230.7 MPa and 219.6 GPa before recovery, and 3103.6 MPa and 214.3 GPa after recovery. A comparison of the tensile properties before and after recovery revealed a significant reduction in the tensile strength of the carbon fiber monofilament after recovery, while the tensile properties of the carbon fiber monofilament before recovery were largely maintained.

The test results are analyzed using a two-parameter Weibull distribution function to characterize the statistical distribution of the tensile properties of the two groups of carbon fibers (before recovery and after recovery). It can be seen from Figure 3b,c that the Weibull distribution plots of the two groups of carbon fibers are approximately linear. The overall tensile strength values of the recovered fibers are smaller and more discrete compared to the virgin fiber tensile.

As depicted in Figure 3d, the Raman spectrum of carbon fibers displays two distinct peaks at approximately 1340 cm^−1^ and 1595 cm^−1^. The D peak corresponds to the vibrational motion of amorphous (disordered) carbon atoms, whereas the G peak represents the lattice vibration associated with the ordered carbon atom structure [20]. The positions of the D and G peaks exhibit minimal variation, indicating little change in the internal composition of the carbon fiber before and after the recovery process. The ratio of the intensities of the D and G peaks, referred to as R(*I_D_/I_G_*), serves as a measure of the level of irregularity in the arrangement of carbon atoms. A lower R value indicates greater crystal integrity of the carbon fiber and a higher proportion of ordered carbon atom structure. The recovered carbon fiber demonstrates more distinct D and G peaks compared to the original carbon fiber prefilaments, along with a slightly higher R-value. This suggests a slight reduction in the integrity of the crystal structure, resulting in a higher level of disorder in the carbon fiber. This observed change may have implications for the mechanical properties of the recovered carbon fiber-reinforced composite, potentially leading to decreased strength and stiffness [21].

The outcomes of the infrared analysis of recovered carbon fiber and carbon fiber filament are presented in Figure 3e. The carbon fiber filament exhibits multiple infrared absorption peaks corresponding to the vibration of a variety of chemical bonds within the material. The peak observed at 3435 cm^−1^ suggests the stretching vibration of the hydroxyl group (O–H), suggesting the potential presence of a significant number of hydrogen bonds on the material’s surface. Peaks at 2969 cm^−1^ and 2856 cm^−1^ correspond to the antisymmetric and symmetric expansion vibration of methyl and methylene, respectively. Additionally, peaks at 1456 cm^−1^ denote the variable angle vibration of methylene and the asymmetric variable angle vibration of methyl, while peaks at 1382 cm^−1^ indicate the symmetric variable angle vibration of methyl. The absorption peak at 1719 cm^−1^ corresponds to the stretching vibration of the carbonyl group (C=O), implying the presence of unsaturated organic compounds or ketones in the material. Furthermore, the peak at 1233 cm^−1^ represents C=O stretching vibration, suggesting the presence of ester or carboxyl functional groups within the material [22]. These infrared absorption peaks provide crucial insights into the chemical composition and structure of carbon fibers prior to recovery, facilitating in the comprehension of the material’s properties and potential reactivity. The recovered carbon fiber exhibits a novel absorption peak at 1586 cm^−1^, corresponding to the skeletal vibration of the aromatic ring. This result suggests the potential development of an aromatic ring structure on the carbon fiber surface as a result of the recovery treatment, potentially augmenting the material’s ability to adsorb organic substances in environmental gases [23]. Furthermore, two additional absorption peaks at 1387 cm^−1^ and 1346 cm^−1^, corresponding to variable angle vibrations of C–H, are detected. This occurrence may be attributed to the formation of new C–H bonds [24], as some hydrogen atoms on the material’s surface bond with carbon atoms during the recovery process. Moreover, the intensified absorption peaks of C–O stretching vibration at 1114 cm^−1^ and 1055 cm^−1^ imply that the recovering process may lead to the formation of C–O bonds, as some carbon atoms on the carbon fiber’s surface combine with oxygen atoms [25]. The formation of C–H and C–O bonds may simultaneously augment the material’s bonding properties and chemical reactivity.

**Figure 4:**
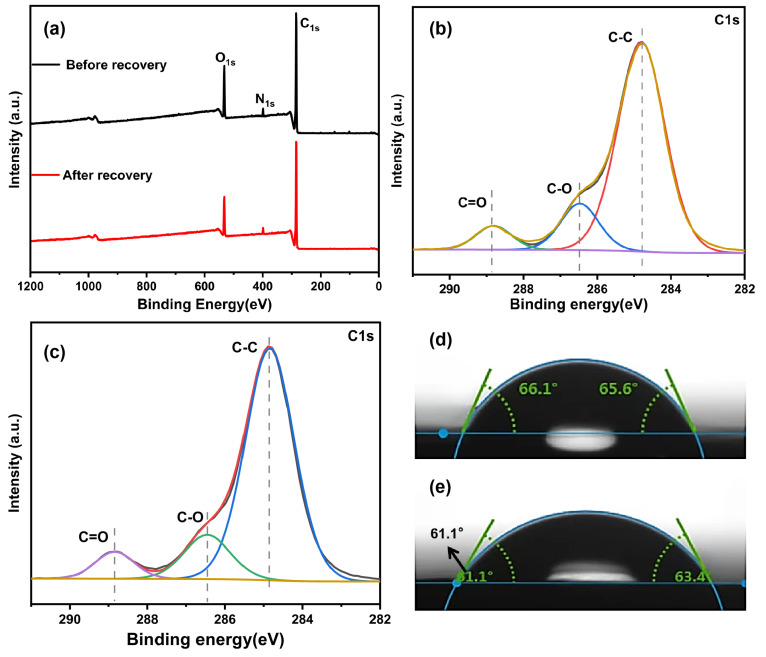
(**a**) X-ray photoelectron spectra of carbon fiber before and after recovery. Fitting curve of C1s peak of carbon fiber (**b**) before recovery and (**c**) after recovery. Contact angle test of carbon fiber (**d**) before recovery and (**e**) after recovery.

The composition and characteristics of functional groups present on the surface of carbon fiber were subsequently examined both before and after the recovery process, as shown in Figure 4a. The contents of the main elements of carbon fiber before and after recovery are illustrated in Table 1. Carbon fiber primarily contains C, O, and N elements before and after recovery. The C 1s peaks observed both before and after recovery comprised 73.8% and 72.22% of the total peak area, respectively, suggesting no significant alteration in the relative carbon content. The O 1s peaks before and after recovery accounted for 11.63% and 13.04% of the total peak area, respectively. This increase indicates a rise in the relative oxygen content and the number of oxygen-containing functional groups to a certain extent. The N 1s peaks before and after recovery accounted for 2.22% and 2.23% of the total peak area, respectively, indicating that the relative nitrogen content remained relatively unchanged.

The Peak C 1s were segmented, and the fitting outcomes are depicted in Figure 4b,c, whereas the functional group composition is exhibited in Table 2. The spectra before and after regeneration exhibited three primary peaks, specifically C 1s at 285 eV, O 1s at 532 eV, and N 1s at 399 eV. The carbon fiber’s surface before and after recovery is primarily composed of inert carbon atoms [26]. After the recovery process, there was a minor modification in the functional groups present on the surface of the carbon fiber. This modification resulted in a small elevation in the carbonyl group content, whereas the hydroxyl and carbonyl groups continued to be the prevalent forms. The XPS test results were essentially consistent with the infrared test findings, implying that the type and quantity of elements remained mostly unchanged, save for minor variations in oxygen-containing functional groups [27]. As a result, the properties of composite materials fabricated utilizing recovered carbon fiber strongly resemble those of the original carbon fiber.

The surface wettability and interface interaction of carbon fiber were assessed by conducting testing to measure the contact angle between carbon fiber and epoxy resin. The test results are shown in Figure 4d,e. In Figure 4d, the contact angles of the short-cut carbon fiber before recovery are 66.1° and 65.6°, respectively, while, in Figure 4e, the contact angles of the short-cut carbon fiber after recovery are 61.1° and 63.4°, respectively, with slightly smaller contact angles [28]. Therefore, the wettability of recovered carbon fiber after recovery treatment can be improved, potentially enhancing the binding performance between carbon fiber and epoxy resin and consequently improving the performance of composite materials.

### 3.2. Mechanical Properties of Composite Plates

Pendulum impact tests were conducted on the carbon fiber-reinforced composite before and after recovery. The pendulum energy of the pendulum impact test was 5 J and the span was 70 mm. The findings, presented in Table 3, indicate that the impact strength of the carbon fiber-reinforced resin matrix composite decreased from 48.83 kJ/m^2^ before recovery to 37.05 kJ/m^2^ after recovery [29]. Additionally, the fracture mode of the composites was a complete ductile fracture in both cases. This comparison clearly demonstrates a reduction in the impact strength of the recovered carbon fiber-reinforced composite.

**Figure 5:**
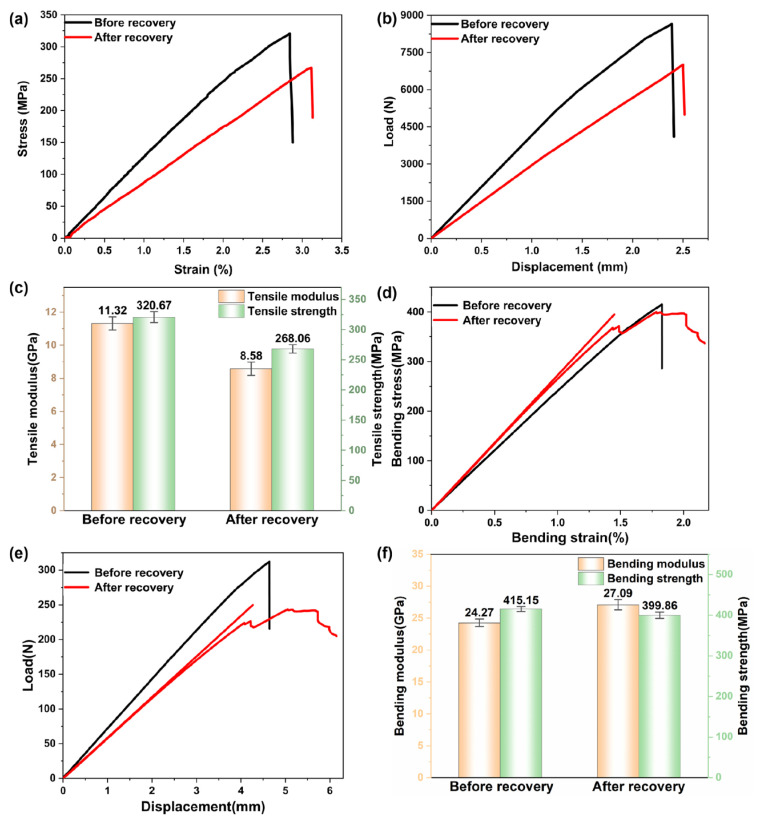
(**a**) Tensile testing stress–strain curve, (**b**) tensile test force–displacement curve, (**c**) tensile test result, (**d**) bending tests stress–strain curves, (**e**) bending test force–displacement curve, and (**f**) bending performance test results.

The carbon fiber-reinforced composite underwent a tensile test, and the stress–strain curves before and after recovery are exhibited in Figure 5a. It can be seen that the failure strain of the before recovery specimen was about 2.8%. The failure strain of the after-recovery specimen was about 3.1%. The force–displacement curves before and after recovery are depicted in Figure 5b. Additionally, the tensile test results of the carbon fiber-reinforced composites before and after recovery are illustrated in Figure 5c. The tensile test results demonstrated that the elastic modulus of the composite dropped from an average of 11.32 GPa before the recovery process to an average of 8.58 GPa after the recovery process. The tensile strength of the recovered carbon fiber-reinforced resin matrix composites also decreased from 320.67 MPa to 268.06 MPa, leading to a decline in the tensile mechanical properties [30].

The carbon fiber-reinforced composite underwent a bending test. The bending strain curves of the composite before and after recovery are presented in Figure 5d, while the force–displacement curves during the bending test are presented in Figure 5e. Additionally, the bending performance test results are illustrated in Figure 5f. Compared with the bending test results before and after recovery, the bending strength of the recovered carbon fiber-reinforced resin matrix composites decreased from an average of 415.15 MPa to an average of 399.86 MPa. The fracture bending strain declined from 2.364% to 2.169%. The fracture bending displacement dropped from 6.352 mm to 6.147 mm [31]. The experimental results reveal that the bending mechanical properties of composites composed of recovered carbon fiber exhibit negligible reduction, and the bending properties of the composites are essentially preserved. The tensile and bending test outcomes demonstrate that there is a huge difference between the tensile and bending modulus, which is because of the different stress states and strain distribution of the material under these two test conditions [32].

**Figure 6:**
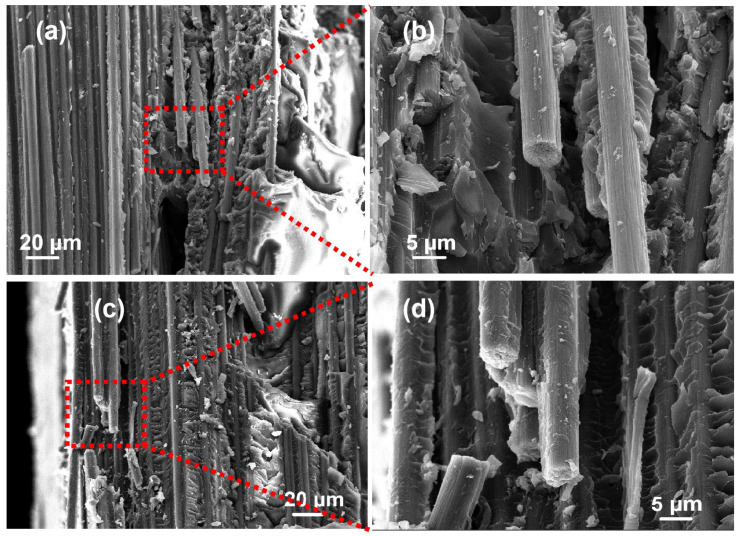
(**a**,**b**) SEM results of carbon plates before recovery and (**c**,**d**) SEM results of recovered carbon plates.

The microstructural characteristics of the carbon fiber-reinforced composite are depicted in Figure 6a,b, while the microstructure of the recovered carbon fiber-reinforced composite is illustrated in Figure 6c,d. The diameter and surface smoothness of the carbon filament only shows negligible changes before and after recovery. The preparation process of the carbon fiber-reinforced composite does not display any discernible alterations before and after the recovery process, thereby facilitating the recovery and reutilization of the recovered carbon fiber materials.

## 4. Conclusions

The research examined the preparation process and properties of resin matrix composites reinforced with recovered carbon fibers. The recovered carbon fiber was obtained using the microwave pyrolyzation–oxidation method. Subsequently, the recovered carbon fiber was mixed with epoxy resin in proportion, and the recovered carbon fiber-reinforced resin matrix composite was processed through hot pressing. The examination revealed that the structural integrity of the recovered carbon fiber remained unchanged, whereas the presence of oxygen-containing functional groups on its surface proliferated. On the other hand, the internal crystal structure integrity declined, resulting in a reduction in its properties. As a result, the mechanical properties of the composite produced from the recovered carbon fiber decreased, including the impact strength, tensile strength, and bending strength, with the impact strength dropping by 24.14%, tensile strength by 15.94%, and bending strength by 8.24%. Even with these reductions, the composites demonstrated satisfactory performance, suggesting their potential reusability in specific applications and fields.

## Figures and Tables

**Table 1:** Type of material element.

	Element Name	Energy/eV	Wavelength/μm	Wave Number/cm^−1^	Cpeak/%
Before recovery	C 1s	285.13	3.01	1,404,777	72.22 ± 0.5
	O 1s	532.75	3.28	612,550.5	13.04 ± 0.2
	N 1s	399.53	1.5	67,448.27	2.23 ± 0.1
After recovery	C 1s	285.12	2.97	1,444,991	73.8 ± 0.3
	O 1s	532.84	3.32	550,435.6	11.63 ± 0.1
	N 1s	399.43	1.72	67,526.24	2.22 ± 0.05

**Table 2:** Peak analysis of C1s of carbon fiber before and after recovery.

Carbon Fiber		Before Recovery	After Recovery
C–C	E/eV	284.8	284.8
	Cpeak/%	78.93	78.65 ± 0.4
C–0	E/eV	286.47	286.46
	Cpeak/%	14.22	14.11 ± 0.3
C=0	E/eV	288.8	288.84
	Cpeak/%	6.85	7.24 ± 0.05

**Table 3:** Results of pendulum impact tests of carbon fiber-reinforced composites.

Index Name	Before Recovery	After Recovery
Width/mm	4.962	4.63
Absorbed energy/J	0.667 ± 0.02	0.41 ± 0.01
Impact strength/(kJ/m^2^)	48.83 ± 0.5	37.05 ± 0.3
Fracture mode	ductile	ductile

## Data Availability

Data will be made available on request.
